# Vaccination Coverage and Associated Factors of COVID-19 Uptake in Adult Primary Health Care Users in Greece

**DOI:** 10.3390/healthcare11030341

**Published:** 2023-01-24

**Authors:** Izolde Bouloukaki, Anna Christoforaki, Antonios Christodoulakis, Thodoris Krasanakis, Eirini Lambraki, Rodanthi Pateli, Manolis Markakis, Ioanna Tsiligianni

**Affiliations:** 1 Health Planning Unit, Department of Social Medicine, School of Medicine, University of Crete, 71500 Heraklion, Greece; 2 Primary Care Health Center of Kastelli, 70006 Heraklion, Greece

**Keywords:** COVID-19 pandemic, vaccination, attitudes, perceptions

## Abstract

In our study, attitudes and perceptions of adult primary health care users regarding COVID-19 vaccination were evaluated. A single-center, cross-sectional study was conducted during a 1-year period (March 2021–March 2022) in a rural area in Crete, Greece. A sample of 626 self-reported questionnaires was collected at the end of the study period. Overall, 78% of respondents stated that they had received the COVID-19 vaccine. The reasons behind vaccine uptake were mainly personal beliefs and the desire to avoid professional constraints. The presence of diabetes type 2, fear of infection, and high perceived efficacy of vaccine previous flu vaccination, living with vulnerable persons, and the influence of scientific information were all significant predictors of COVID-19 vaccine uptake. On the contrary, unwillingness and/or uncertainty to be vaccinated was associated with fear of vaccine side effects, information insufficiency, media/internet information, older age, the presence of inflammatory arthritis, previous COVID-19 infection, the belief that infection confers much greater immunity than the vaccine, and attitudes against vaccinations in general were predictors against COVID-19 vaccination. In conclusion, taking into account all of the above predictors and particularly those regarding safety and vaccine effectiveness may guide future strategies appropriately tailored to specific characteristics and needs of different geographic populations.

## 1. Introduction

COVID-19 and the subsequent pandemic were first reported by the World Health Organization (WHO) about two years ago; however, humanity continues to deal with its significant health, social, and economic repercussions [1]. As of 7 October 2022, over 600 million confirmed cases and over six million deaths have been reported globally [2]. The only way of returning to normality appears to be the development of herd immunity through natural infection or vaccination [3].

Massive immunization against COVID-19 is likely the most powerful and cost-effective health intervention, as well as the most promising strategy to control the COVID-19 pandemic, as opposed to lockdown measures or facemask use, which are both emotionally and physically challenging. It is estimated that reaching 70% immunization coverage can further control the epidemic [4]. However, while vaccination uptake was initially dependent on supply issues, there have been reports of significant vaccine "hesitancy" affecting current vaccine uptake [5], especially in various ethnic groups around the globe, resulting in much lower than expected immunization rates [6,7,8,9,10,11]. Globally, and even among healthcare workers, studies show a mixed response to COVID-19 vaccine acceptance [12,13,14,15,16,17].

A number of variables contributing to vaccine hesitancy arise from concerns of confidence (do not trust the vaccine or the provider), complacency (do not recognize a need for a vaccination, do not value the vaccine), and convenience (access) [18]. Moreover, the rapid vaccine development, fear of possible side effects and complications, influence of friends, family and conspiracy theories, as well as inaccurate information broadcast via the media seemed to be major concerns against vaccination [5,19,20]. Importantly, vaccine hesitation and refusal seem to be closely linked to health literacy, perspectives, knowledge, and attitudes [21,22]. On the other hand, COVID-19 risk perception and certain sociodemographic traits (i.e., being male, young, and having children under the age of 18) were shown to be strongly linked with vaccination uptake [23]. Trust in health officials, as well as residing in urban regions and having a college education (as opposed to having a primary or secondary education), were also positively associated with COVID-19 vaccination intention [24]. Vaccination for protection against COVID-19 and return to normality should also be considered.

Vaccinations for COVID-19 began in Greece in late December 2020, and started with people being at the front lines of health care, for vulnerable groups, those in closed structures/care structures, and for those who serve in important state services, and then was expanded to all adults. From May 2021, the Greek authorities gradually introduced mandatory vaccination for certain professions (e.g., in healthcare or public services) and age groups (over 60 years), excluding those who had recently recovered from COVID-19 or had a medical contraindication to vaccination. After imposing compulsory vaccination on the above categories, the Joint Ministerial Decision of 24 October 2021 went a step further, allowing access to indoor catering and entertainment businesses and then to retail businesses exclusively to citizens who were fully vaccinated or who had recovered from COVID-19 in the last six months [25]. These policies appear to have resulted in Greece having higher vaccination intention rates among its population when compared to other European countries [26]. Indeed, by the end of December 2022, 77% of the total population had received at least one vaccine dose, and 72% were fully vaccinated [27].

However, there are not many studies which have examined the attitudes and perceptions of COVID-19 vaccination in adult primary health care users in Greece [3,26,28,29,30,31,32,33]. Understanding their perceptions and their willingness to be vaccinated are crucial for improving vaccination rates and the taking of booster shots. Therefore, the aim of the present study was to investigate predictors, including attitudes and perceptions that influence the decision to get COVID-19 vaccination in adult primary health care users of a rural area in Crete, Greece.

## 2. Materials and Methods

### 2.1. Study Setting and Participants

In this single-center, cross-sectional study, consecutive patients aged ≥ 18 years were approached by general practitioners (GPs) during a regular consultation in our primary health care center in Crete, during a 1-year period (March 2021–March 2022). Eligible for the study were those who were at least 18 years of age and who had provided informed consent. Exclusion criteria included patients with any severe neurological or mental disease or poor understanding of the Greek language. Ethical approval was provided by the Primary Health Care Centre of Kastelli Scientific Board Ethics Committee (protocol number 18296–3862/28-01-2021), and the patients provided written informed consent.

### 2.2. Study Tool and Administration

Based on previous literature and semi-structured interviews with a convenience sample of 10 patients, a structured questionnaire was developed that was pretested in a convenience sample of 10 primary care physicians and 20 patients. It included questions in the following categories: (1) Socio-demographic characteristics (age, gender, marital status, years of education); (2) Health status factors (smoking status, comorbidities, self-rated health status, vulnerability status and family members’ vulnerability); (3) Attitudes and beliefs about COVID-19 vaccination (17 items with questions of agreement or disagreement); (4) Level of information and questions about the degree of influence of parameters related to vaccine uptake (religion, politics, science, media and internet and anti-vaccine movement), and whether they were vaccinated for influenza (high risk patients) during the last season; and (5) Concerns about getting COVID-19.

All participants were informed about the aims of the study by their primary care physician, and if they consented to participate they provided their written informed consent and completed the questionnaire. No compensation was provided for their participation. Participants were requested to place their completed study tools in a box positioned outside the practice to minimize social desirability bias. 

### 2.3. Statistical Analysis

Results are presented as mean ± standard deviation (SD) for continuous variables if normally distributed, and as median (25th–75th percentile) if not. Qualitative variables are presented as absolute number (percentage). For comparisons between vaccinated and unvaccinated groups, a two-tailed t-test for independent samples (for normally distributed data) or a Mann–Whitney U test (for non-normally distributed data) was utilized for continuous variables, and the Pearson’s chi-square test was used for categorical variables. Correlation coefficients were calculated using the Pearson or Spearman’s (for non-normally distributed data) correlation test for all of the independent predictors of COVID-19 vaccine uptake. As independent variables, we included clinically relevant ones, such as sociodemographic factors (age, gender, marital status, level of education), health related factors (smoking status, co-morbidities, self-rated health status) and factors associated with knowledge, attitudes and beliefs about vaccination. Only the variables that were found to be significant (*p* < 0.05) were further analyzed. A multivariate logistic regression analysis was applied to examine the effect of the significant variables on the vaccine uptake, after controlling for potential explanatory variables, including age, gender, smoking status, education level and co-morbidities. We checked multicollinearity among the predictors using collinearity statistics to ensure that collinearity between predictor variables was in the acceptable range as indicated by the tolerance value variance inflation factor. Age was considered continuously and categorically, as age groups of 18–49, 50–64 and ≥65 years. To define the high-risk groups for the influenza vaccine, we used the criteria presented in the National Immunization Program 2018–19 [34], including age and chronic diseases or risk factors in the patients’ medical history that were collected by the study tool. Results were considered significant when *p* values were <0.05. Data were analyzed using SPSS software (version 25, SPSS Inc., Chicago, IL, USA).

## 3. Results

### 3.1. Study Population

The study population included 626 participants (82% response rate), of whom 264 (42%) were men. The age of participants ranged from 18 to 93 years; 426 (68%) were aged 18–49, 119 (19%) were aged 50–64 and 81 (13%) were aged ≥ 65 years. About 15% of patients had a primary school level education or less. Regarding chronic diseases, 100 patients (18%) had at least one chronic disease. The socio-demographic characteristics and health status factors of the 626 participants are further described in Table 1. Interestingly, most of the participants [513 (82%)] rated their health as good/excellent.

### 3.2. Coverage, Associated Factors, and Level of Awareness per Vaccination

Overall, 493 out of 626 subjects (78%) had received the COVID-19 vaccine. The baseline characteristics of study population, compared according to vaccination status, are shown in Table 1. Vaccinated subjects were significantly younger (41 vs. 46, *p* = 0.01). There were also differences in the way the comorbidities presented. However, only the presence of inflammatory arthritis was significantly higher in the non-vaccinated group (6 vs. 2%, *p* = 0.04). Moreover, the two groups did not differ significantly in terms of gender, level of education, marital status, smoking status or self-rated health status.

Table 2 shows COVID-19 coverage by sex and age stratification. COVID-19 coverage largely differed with increasing age (82% in 18–49 vs. 68% in 50–64 years vs. 75% in ≥65 years; *p* = 0.003), but did not differ significantly between men and women in the whole sample (80% vs. 78%; *p* = 0.58) and in all age groups (all *p* > 0.05).

As shown in Table 3, most of the respondents that had received the COVID-19 vaccine reported that they had taken into account their own personal beliefs (71%) about the COVID-19 vaccine. However, a considerable percentage of subjects stated that professional constraints (22%) were the most important factor.

### 3.3. Knowledge, Attitudes and Βeliefs about COVID-19 Vaccination

A comparison of knowledge, attitudes and beliefs about COVID-19 vaccination between COVID-19 vaccinated and unvaccinated persons is presented in Table 4. Participants vaccinated for COVID-19 more commonly agreed that vaccination would effectively control and prevent COVID-19, feared disease side effects, and were more commonly vaccinated for influenza. Regarding hesitancy in vaccine uptake, unvaccinated participants were significantly more likely to express that lack of information, fear of vaccine side effects, early vaccine distribution, previous COVID-19 infection, the belief that infection confers much greater immunity than the vaccine, and being against vaccinations in general were important factors for deciding whether they would receive the vaccine.

### 3.4. Correlation of COVID-19 Vaccine Uptake with Demographic, Health Status, Knowledge, Attitudes and Βeliefs and Other Sources of Information about COVID-19 Vaccination

Across all subjects (Supplementary Table S1–S3), scientific information (*r* = 0.521, *p* < 0.001), high perceived efficacy of the vaccine (*r* = 0.151, *p* < 0.001), younger age (r = −0.14, *p* < 0.001), previous flu vaccination (*r* = 0.136, p = 0.001), trust in government *(r* = 0.104, *p* = 0.026), fear of infection (*r* = 0.101, *p* = 0.019), being a vulnerable person (*r* = 0.09, *p* = 0.038) or living with a vulnerable person *(r* = 0.09, *p* = 0.029), and the presence of diabetes (*r* = 0.08, *p* = 0.043) were positively correlated with the decision to be vaccinated for COVID-19. On the contrary, media/internet information (*r* = −0.593, *p* < 0.001), early vaccine distribution (*r* = −0.225, *p* < 0.001), the belief that infection confers much greater immunity than the vaccine (*r* = −0.218, *p* < 0.001), lack of information (*r* = −0.212, *p* < 0.001), fear of vaccine side effects (*r* = −0.167, *p* < 0.001), previous COVID-19 infection (*r* = −0.152, *p* < 0.001), against vaccinations in general (*r* = −0.139, *p* = 0.001) and presence of inflammatory arthritis (*r* = −0.08, *p* = 0.036) were negatively correlated with vaccine uptake. Only the variables that were found to be significant (*p* < 0.05) were analyzed further.

Table 5 shows a multivariable-adjusted analysis estimating ORs for COVID-19 vaccine uptake in 516 adult primary health care users with full data, after adjustment for age, gender, smoking status, education level and co-morbidities. Lack of information, fear of vaccine side effects, early vaccine distribution, and media/internet information were still the strongest predictors against COVID-19 vaccination, followed by older age, the presence of inflammatory arthritis, previous COVID-19 infection, belief that infection confers much greater immunity than the vaccine, and against vaccinations in general. On the other hand, the presence of diabetes type 2, fear of infection, previous flu vaccination (in high risk groups), living with vulnerable persons, and scientific information along with high perceived efficacy of the vaccine were in favor COVID-19 uptake. There was no evidence of collinearity in the model, with all variance inflation factors under 1.7 (data not shown).

## 4. Discussion

This single-center, cross-sectional study assessed the prevalence and related factors of acceptance and potential drivers of people’s decision-making in COVID-19 vaccine uptake among an adult primary health care population recruited from a rural area in Crete, Greece. We found that a significant proportion of people got vaccinated for a variety of reasons, the majority of which were related to their personal beliefs and the desire to avoid professional constraints. Importantly, the presence of type 2 diabetes, fear of infection, high perceived efficacy of the vaccine, previous flu vaccination (in high risk groups), and scientific influence were all significant predictors of COVID-19 vaccine uptake. On the contrary, unwillingness and/or uncertainty to be vaccinated was associated with fear of vaccine side effects, information insufficiency, media/internet information, older age, the presence of inflammatory arthritis, previous COVID-19 infection, belief that infection confers much greater immunity than the vaccine, and attitudes against vaccinations in general were predictors against COVID-19 vaccination, suggesting the need to better tailor pandemic response strategies.

Since the pandemic imposes a substantial disease burden on health systems and a threat to global health, massive immunization is regarded as the most effective and cost-effective health intervention, besides the prophylactic community measures [35]. The acceptability of a vaccine is the key determinant of the overall success of vaccination programs taking into account that development and distribution of a new vaccine is an expensive and time-consuming procedure [36]. Therefore, estimating and investigating the common causes of COVID-19 vaccine reluctance is a crucial step in developing an action plan for increasing overall acceptance.

A wide range of COVID-19 vaccine acceptance rates has been reported all over the world [12,36]. Vaccinations for COVID-19 began in Greece in late December 2020, and by the end of December 2022, 77% of the total population had received at least one vaccine dose [27], in accordance with our results. In our population, the high perceived efficacy of the vaccine, previous flu vaccination (in high risk groups), living with vulnerable persons and the influence of scientific information were identified as the most common predictors of COVID-19 vaccine acceptance. Several studies in previous vaccination programs have found that perceived vaccine safety and effectiveness were also the most common factors associated with vaccine hesitancy [37,38,39]. Furthermore, recent systematic reviews and meta-analyses showed that the extent to which the public believes the vaccine is safe and effective after administration, the higher perceived risk of getting infected, and being vaccinated against influenza were indeed among the strongest predictors of COVID-19 vaccine uptake intention [36,40]. It is also worth noting that the association between influenza vaccination and vaccination for COVID-19 was recently confirmed in a recent study in a Greek population [41].

Individual vaccination acceptance is influenced not only by knowledge of vaccine risks and benefits, but also by religious, cultural, emotional, and social factors, which are considered more complex determinants [42,43]. In our study, fear of vaccine side effects, media/internet information, information insufficiency, older age, the presence of inflammatory arthritis, previous COVID-19 infection, the belief that infection confers much greater immunity than the vaccine, and attitudes against vaccinations in general were important negative factors for COVID-19 vaccination. It is well known that hidden and inadequate health information may accelerate anti-vaccine conspiracy beliefs and rumours, interfering in the implementation of successful vaccination programs in different countries [44,45,46]. Moreover, some of the most relevant predictors of COVID-19 vaccine hesitancy identified in our study are likely to be explained by fake news or the spread of imprecise or false information about COVID-19 on social media [47,48,49].

Taking into account all of the above predictors, and particularly that vaccine confidence levels regarding safety and effectiveness are influenced by the level of trust in the vaccine, restoring public trust in vaccines and the vaccination process could be a key solution to vaccine efficacy misconceptions and mistrust. This suggests that during medical consultations, health professionals should assess patients’ perceptions of COVID-19 and COVID-19 vaccination to aid in the immunization decision-making process. As medical professionals play a significant role in improving people’s knowledge, attitudes, and practices regarding the COVID-19 vaccine, they should ideally be trained in communication strategies in order to effectively convey all of these health messages. Furthermore, as media, applications, video games, and social networks can provide high-quality information on the risk and severity of being infected with COVID-19, as well as the safety of COVID-19 vaccines, health communication strategies using high-quality health-related information about COVID-19 vaccines and immunisation could play a role in understanding and reducing vaccine concerns. Such efforts may contribute to the strengthening of long-term trust and the acceleration of COVID-19 vaccination progress.

It is plausible that several limitations might have affected our results. First, given the cross-sectional design of our study, we cannot attribute directionality to the aforementioned associations between COVID-19 vaccination and the various predictive factors. Second, our study was a single center study limiting the generalizability of our findings. Nevertheless, we recruited participants from a representative rural area in Crete. Third, response biases could be another limitation because the data were self-reported. Moreover, as participants’ attitudes toward COVID-19 vaccination may change over time, more research is also needed to monitor changes in vaccine uptake predictive factors over time. 

## 5. Conclusions

In conclusion, it is critical to promote COVID-19 vaccination, and vaccination hesitancy must be mitigated. The above predictors, and particularly those regarding safety and vaccine effectiveness, may guide future strategies at improving vaccine uptake that are appropriately tailored to the specific characteristics and needs of different geographic populations.

## Figures and Tables

**Table 1:** Characteristics of the 626 participants.

	Total Population (*n* = 626)	COVID-19 Unvaccinated (*n* = 133)	COVID-19 Vaccinated (*n* = 493)	*p*-Value
**Demographics**				
Gender, males (%)	264 (42%)	53 (40%)	211 (43%)	0.58
Age, years	42 ± 15	46 ± 17	41 ± 17	0.01
Age ≥ 60 years	106 (17%)	29 (22%)	74 (15%)	0.06
**Level of education**				
Primary level or less	94 (15%)	25 (19%)	69 (14%)	0.07
Secondary level or higher	532 (85%)	108 (81%)	424 (86%)	
**Marital Status**				
Married	357 (57%)	84 (63%)	271 (55%)	0.09
Single	269 (43%)	49 (37%)	222 (45%)	
**Smoking status**				
Current	244 (39%)	49 (37%)	197 (40%)	0.60
Never/Former smoker	382 (61%)	84 (63%)	296 (60%)	
**Co-morbidities**				
COPD	10 (2%)	1 (1%)	9 (2%)	0.39
Asthma	23 (4%)	6 (5%)	17 (3%)	0.55
Diabetes Type 2	22 (4%)	1 (1%)	21 (4%)	0.05
Coronary artery disease	15 (2%)	6 (5%)	9 (2%)	0.07
Stroke/TIA	4(1%)	2 (2%)	2 (1%)	0.16
Cancer	12 (2%)	4 (3%)	8 (2%)	0.30
AF	11 (2%)	1 (1%)	10 (2%)	0.32
Inflammatory arthritis	20 (3%)	8 (6%)	12 (2%)	0.04
**Self-rated health**				
Very poor/poor	13 (2%)	3 (2%)	10 (2%)	0.75

Data are presented as mean values ± SD or median (25th–75th percentile), unless otherwise indicated. COPD, Chronic obstructive pulmonary disease, TIA, transient ischemic attack, AF, Atrial fibrillation.

**Table 2:** COVID-19 vaccination coverage by sex and age stratification.

Characteristics	COVID-19 Unvaccinated *n* = 133	COVID-19 Vaccinated *n* = 493	*p*-Value
**Age range, years**			
**All ages**			
All	133/626 (21%)	493/626 (79%)	
Males	53/264 (20%)	211/264 (80%)	
Females	79/362 (22%)	283/362 (78%)	0.58
**18–49**			
All	77/426 (18%) ^*^	349/426 (82%) ^*^	
Males	35/180 (19%)	145/180 (81%)	
Females	44/246 (18%)	202/246 (82%)	0.78
**50–64**			
All	38/119 (32%) ^**^	81/119 (68%) ^**^	
Males	15/47 (32%)	32/47 (68%)	
Females	23/72 (32%)	49/72 (68%)	0.98
**≥65**			
All	20/82 (25%)	61/81 (75%)	
Males	5/35 (16%)	30/35 (84%)	
Females	15 (33%)	31/46 (67%)	0.09

^*^ *p* < 0.001; 18–49 vs. 50–64 and ≥65 years age groups, ^**^ *p* < 0.001; 50–64 vs. 18–49 and ≥65 years age groups.

**Table 3:** Reported sources of information influencing COVID-19 uptake.

Factors	COVID Vaccine No. (%) (Total 493)
**Personal beliefs**	350 (71%)
**Doctors recommendation**	79 (16%)
**Media/Internet**	15 (3%)
**Professional constraints**	99 (20%)

**Table 4:** Reported knowledge, attitudes and beliefs about COVID-19 for vaccinated and for unvaccinated subjects.

Characteristics	Total Population (*n* = 626)	Unvaccinated (*n* = 133)	Vaccinated (*n* = 493)	*p*-Value
Information insufficiency	100 (16%)	36 (27%)	64 (13%)	<0.001
Fear of vaccine side effects	413 (66%)	106 (80%)	307 (62%)	<0.001
Conspiracy beliefs	14 (2%)	5 (4%)	9 (2%)	0.17
High perceived efficacy of vaccine	62 (10%)	3 (2%)	59 (12%)	<0.001
Early vaccine distribution	120 (19%)	41 (28%)	79 (16%)	<0.001
Pregnancy	22 (4%)	3 (2%)	19 (4%)	0.250
No need due to previous COVID-19 infection	22 (4%)	12 (9%)	10 (2%)	<0.001
Belief that infection confers much greater immunity than a vaccine	18 (3%)	13(10%)	5 (1%)	<0.001
Perception of low susceptibility to disease or possible infection would not be severe	23 (4%)	8 (6%)	15 (3%)	0.147
Family influence	21 (3%)	6 (5%)	15 (3%)	0.304
Belief that vaccine development is a way for pharmaceutical companies to make a profit	18 (3%)	3 (2%)	15 (3%)	0.772
Against vaccinations in general	18 (3%)	9 (7%)	9 (2%)	0.001
Fear of infection	361 (57%)	65 (49%)	296 (60%)	0.019
Previous flu vaccination (in high risk population *n* = 147)	93/147 (63%)	18/46 (39%)	75/101 (74%)	<0.001

**Table 5:** Multivariate logistic regression analysis of factors associated with COVID-19 vaccine uptake (*n* = 516).

Multivariate Logistic Regression Analysis		
Variables	Adjusted OR (95% CI)	*p*-Value
**Sociodemographic factors**		
Age	0.98 (0.96–0.99)	0.004
**Health status factors**		
Diabetes type 2	11.27 (1.44–88.03)	0.02
Inflammatory Arthritis	0.26 (0.08–0.82)	0.02
**Knowledge, Attitudes and Βeliefs about COVID-19 vaccination**		
Information insufficiency	0.058 (0.014–0.242)	<0.001
Fear of vaccine side effects	0.39 (0.23–0.64)	<0.001
High perceived efficacy of vaccine	6.0 (1.81–19.93)	0.003
Early vaccine distribution	0.08 (0.026–0.275)	<0.001
No need due to previous COVID-19 infection	0.18 (0.06–0.49)	0.001
Belief that infection confers much greater immunity than a vaccine	0.15 (0.05–0.49)	0.002
Against vaccinations in general	0.22 (0.07–0.68)	0.008
Fear of infection	1.67 (1.08–2.57)	0.02
Previous flu vaccination (in high risk population)	4.96 (1.93–12.67)	0.001
Vulnerable group	0.79 (0.36–1.79)	0.58
Living with vulnerable groups	1.88 (1.17–3.03)	0.009
**Other sources of information**		
Trust in government	2.8 (0.62–12.7)	0.18
Science opinion	22.36 (11.71–42.71)	<0.001
Media/internet	0.04 (0.02–0.08)	<0.001

## Data Availability

The datasets generated during and/or analysed during the current study are available from the corresponding author on reasonable request.

## Supplementary Materials

The following supporting information can be downloaded at: https://www.mdpi.com/article/10.3390/healthcare11030341/s1, Table S1. Correlation of demographic and health status factors parameters with COVID-19 vaccination uptake. Table S2. Correlation of Knowledge, Attitudes and Βeliefs about COVID-19 vaccination with COVID-19 vaccination uptake. Table S3. Correlation of other sources of information about COVID-19 vaccination with COVID-19 vaccination uptake.
