# Supplementary material for: Vaccination Coverage and Associated Factors of COVID-19 Uptake in Adult Primary Health Care Users in Greece
Source: Healthcare (Basel). 2023 Jan 24;11(3):341. doi: 10.3390/healthcare11030341 (PMC9914444; doi:10.3390/healthcare11030341)
Supplement: Supplementary file 1 [file healthcare-11-00341-s001.zip › healthcare-2162501-supplementary.pdf]

SUPPL. TABLES

Table S1. Correlation of demographic and health status factors parameters with COVID-19 vaccination uptake.

**Parameters**	**Decision to vaccinate**	
	**r Value**	**P Value**
Age	**-0.14**	**<0.001**
Females versus males	-0.022	0.585
Married vs Single	-0.08	0.094
Lower education (≤6 years)	-0.07	0.139
**Health status factors**		
Current Smoking	0.02	0.601
Comorbidities (≥1)	-0.07	0.082
Comorbidities (≥2)	0.10	0.800
COPD	0.04	0.386
Asthma	-0.02	0.553
Diabetes type 2	**0.08**	**0.043**
Coronary Artery Disease	-0.07	0.070
Stroke/TIA	-0.06	0.156
Cancer	-0.04	0.296
Inflammatory Arthritis	**-0.08**	**0.036**
Low self-rated health	0.029	0.479

Table S2. Correlation of Knowledge, Attitudes and Beliefs about COVID-19 vaccination with COVID-19 vaccination uptake.

**Parameters**	**Decision to vaccinate**	
**Knowledge, Attitudes and Beliefs about COVID-19 vaccination**	**r Value**	**P Value**
Information insufficiency	**-0.212**	**<0.001**
Fear of vaccine side effects	**-0.167**	**<0.001**
Conspiracy beliefs	-0.06	0.169
High perceived efficacy of vaccine	**0.151**	**<0.001**
Early vaccine distribution	**-0.225**	**<0.001**
Pregnancy	0.05	0.251
No need due to previous COVID-19 infection	**-0.152**	**<0.001**
Belief that infection confers much greater immunity than a vaccine	**-0.218**	**<0.001**
Perception of low susceptibility to disease or possible infection would not be severe	-0.06	0.148
Family influence	-0.044	0.305
Belief that vaccines development is a way for pharmaceutical companies to make profit	0.013	0.773
Against vaccinations in general	**-0.139**	**0.001**
Fear of infection	**0.101**	**0.019**
Previous flu vaccination (in high risk population)	**0.136**	**0.001**
Vulnerable group	**0.09**	**0.038**
Living with vulnerable groups	**0.09**	**0.029**

Table S3. Correlation of other sources of information about COVID-19 vaccination with COVID-19 vaccination uptake.

**Parameters**	**Decision to vaccinate**	
**Other sources of information**	**r Value**	**P Value**
Religion	0.007	0.881
Trust in government	**0.104**	**0.026**
Science opinion	**0.521**	**<0.001**
Media/internet	**-0.593**	**<0.001**
Anti-vaccination campaign	-0.07	0.107
